# The Regulation of Translation in Alphavirus-Infected Cells

**DOI:** 10.3390/v10020070

**Published:** 2018-02-08

**Authors:** Luis Carrasco, Miguel Angel Sanz, Esther González-Almela

**Affiliations:** Centro de Biología Molecular Severo Ochoa (CSIC-UAM), Universidad Autónoma de Madrid c/Nicolás Cabrera, 1., Cantoblanco, 28049 Madrid, Spain; masanz@cbm.csic.es (M.A.S.); emgonzalez@cbm.csic.es (E.G.-A.)

**Keywords:** regulation of translation, alphaviruses, initiation factors, RNA structure, IRES

## Abstract

Sindbis virus (SINV) contains an RNA genome of positive polarity with two open reading frames (ORFs). The first ORF is translated from the genomic RNA (gRNA), rendering the viral non-structural proteins, whereas the second ORF is translated from a subgenomic mRNA (sgRNA), which directs the synthesis of viral structural proteins. SINV infection strongly inhibits host cell translation through a variety of different mechanisms, including the phosphorylation of the eukaryotic initiation factor eIF2α and the redistribution of cellular proteins from the nucleus to the cytoplasm. A number of motifs have been identified in SINV sgRNA, including a hairpin downstream of the AUG initiation codon, which is involved in the translatability of the viral sgRNA when eIF2 is inactivated. Moreover, a 3′-UTR motif containing three stem-loop structures is involved in the enhancement of translation in insect cells, but not in mammalian cells. Accordingly, SINV sgRNA has evolved several structures to efficiently compete for the cellular translational machinery. Mechanistically, sgRNA translation involves scanning of the 5′-UTR following a non-canonical mode and without the requirement for several initiation factors. Indeed, sgRNA-directed polypeptide synthesis occurs even after eIF4G cleavage or inactivation of eIF4A by selective inhibitors. Remarkably, eIF2α phosphorylation does not hamper sgRNA translation during the late phase of SINV infection. SINV sgRNA thus constitutes a unique model of a capped viral mRNA that is efficiently translated in the absence of several canonical initiation factors. The present review will mainly focus in the non-canonical mechanism of translation of SINV sgRNA.

## 1. Introduction

Sindbis virus (SINV) belongs to the alphavirus genus in the *Togaviridae* family and contains a positive-strand RNA genome [1]. The alphavirus genus comprises ~30 virus species that are transmitted by arthropods, typically mosquitoes, to a range of vertebrate hosts [2]. Exceptions to this rule are the aquatic viruses salmonid alphavirus and Southern elephant seal virus, which are not transmitted by mosquitoes. In addition, Eilat virus can replicate only in insects [3]. Alphaviruses and their genetic variants have a very broad geographical distribution, indicating an ancient origin and evolution [4,5]. Alphaviruses can be subdivided into two groups according to their geographical origin—Old World viruses and New World viruses. Examples of Old World alphaviruses include Semliki Forest Virus (SFV), Chikungunya virus (CHIKV), Ross River virus (RRV) and O’nyong’nyong virus (ONNV), whereas New World alphaviruses are represented by SINV, Venezuelan, Western and Eastern equine encephalitis viruses (VEEV, WEEV and EEEV) [2]. In mammals, alphaviruses typically cause an acute infection, leading to a variety of symptoms and illnesses that are dependent on the virus and host [1], including encephalitis, polyarthritis, myalgia, athritis and rash. By contrast, insects survive the acute phase of infection and become persistently infected for life without apparent pathological consequences [6,7]. Although, some mosquito cells infected with SINV can die in culture in a cell clone specific manner [6]. SINV and SFV have been widely used in the laboratory as model systems to study protein synthesis, transcription and replication at the molecular level, and to understand viral pathogenesis and the interaction of these viruses with their hosts. In this regard, fundamental aspects of translation regulation in virus-infected cells have been uncovered using SINV and SFV. Moreover, the mechanisms of protein synthesis directed by SINV mRNAs are helping to shed light on the structure-function relationship of viral mRNAs. From a practical viewpoint, SINV has been employed in fields as diverse as cancer therapy and has aided in the understanding of the adaptive antiviral response [8,9]. In the current review, we will summarize what is known about the mechanisms of translation of SINV mRNAs, with a focus on the initiation events of non-canonical translation of subgenomic mRNA (sgRNA).

## 2. Overview of the Sindbis Virus Life Cycle

**Figure 1:**
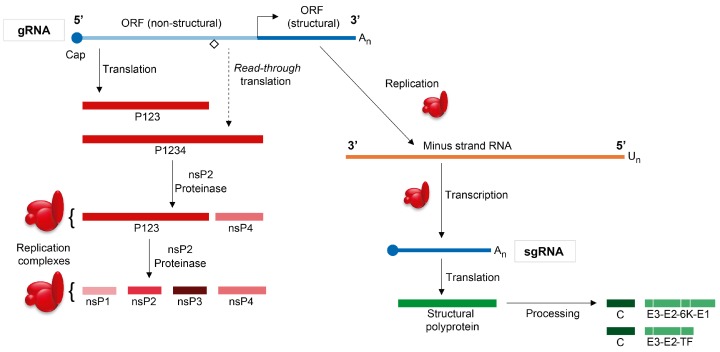
Schematic representation of the synthesis of SINV non-structural and structural proteins. SINV has two different mRNAs that are translated at different times during infection. SINV genomic RNA (gRNA) codes both for non-structural proteins (nsPs) and structural proteins. The first two thirds of the SINV genome is translated to nsP1–nsP4, which are required for transcription and replication of SINV RNA; the remaining one-third of the genome codes for the viral structural proteins. This subgenomic mRNA (sgRNA) is transcribed from an internal promoter in the minus strand RNA derived from the replication of the gRNA, and is translated to a polyprotein that will be processed to C (capsid)-E3-E2-6K-E1. ORF: open reading frame.

The SINV virion is approximately 70 nm in diameter and has a single-strand 11.7 kb RNA genome contained within an icosahedral-structured nucleocapsid made up of 240 copies of capsid protein [10,11]. This is enveloped by a host-derived lipid bilayer membrane into which are embedded viral-encoded glycoproteins E1 and E2. SINV replication occurs in the cytoplasm of infected cells and begins by the recognition of receptors at the cell surface. These receptors include the laminin receptor in mammalian cells, the C-type lectins DC-SIGN and L-SIGN in dendritic cells and the metal ion transporter NRAMP (Natural Resistance-Associated Macrophage Protein), expressed both in mammalian and insect hosts [12,13,14]. After entry, virus particles can follow different pathways to reach the cytoplasm, the most relevant of which is the endocytosis mediated by clathrin. Following endocytosis, virions are delivered into acidic endosomes from which, after fusion of the virus and endosome membranes, the positive-sense RNA genome is delivered into the cytoplasm [15,16,17]. Virions can also enter cells by directly penetrating the plasma membrane [18]. Efficient infection requires that the genome maintains interactions with the capsid protein after genome delivery to the cytoplasm [19]. The arrival of the SINV genome RNA to the cytoplasm can specifically activate the protein kinase general control nonderepressible-2 (GCN2), triggering an early antiviral response [20]. The SINV genome contains two open reading frames (ORFs) that are expressed from two different mRNAs that are translated at different times during the infection process––the genomic RNA (gRNA) and the sgRNA (Figure 1). The gRNA comprises the proximal two-thirds of the genome at the 5′ end, and serves as mRNA for the synthesis of non-structural proteins (nsPs), whereas the more distal one-third sgRNA encodes for structural proteins (Figure 1). The gRNA is translated promptly after virus entry and genome delivery, whereas the sgRNA is translated at late phases of infection [21]. Both gRNA and sgRNA are capped at their 5′ ends and contain a poly(A) tail at the 3′ end. Interestingly, a portion of gRNAs do not contain a cap structure at their 5′ end [22]. The first event in SINV replication is the translation of the imput gRNA to produce nsP1–4, which participate in genome replication and transcription [11]. These nsPs are synthesized from a single AUG initiation codon initially producing two precursor polyproteins (P123 and P1234), which are then post-translationally processed through proteolytic cleavage by nsP2 [23,24,25,26] (Figure 1). After initiation at the first AUG initiation codon in gRNA, the majority of ribosomes (90–95%) translate this template until a stop codon (UGA) is encountered, producing the first of the two precursor polyproteins, P123 [27]. In a small proportion of cases, however, there is read-through of this stop codon, which can be suppressed by several aminoacyl-tRNAs, generating the second precursor polyprotein, P1234. The precise function of the individual nsPs has been the subject of intensive research [24,25,26,28]. nsP1 is a palmitoylated protein that comprises an abundant component of the replicative complex and can interact with membranes. It functions in the initiation and elongation of the minus-strand RNA synthesis via its interaction with nsP4 [29,30]. The N-terminal moiety of nsP1 exhibits methyltransferase and guanylyltransferase activities, which are involved in capping the viral positive-strand RNAs [31,32,33]. Its association with cellular membranes is promoted by an amphipatic helix located in the middle region of nsP1 [33], which serves to anchor viral replicative complexes to membranes [34]. In addition, nsP1 can exhibit its activity either as a mature protein or in the form of the precursors P123 or P1234. nsP2 also contains several domains––an amino-terminal RNA helicase domain, a central protease region that catalyzes all cleavage reactions between the non-structural proteins, and an inactive RNA methyltransferase-like moiety [35,36]. nsP2 also functions in the obstruction of host cellular macromolecular synthesis, such as transcription and translation, and can accordingly antagonize cellular antiviral responses triggered by alphavirus infection [37,38]. Indeed, a fraction of nsP2 localizes to the nucleus and blocks cellular RNA export to the cytoplasm [38,39]. Moreover, nsP2 induces degradation of Rpb1, a catalytic subunit of the RNA polymerase II polymerization complex, mediated by its ubiquitination [40]. nsP3 is also organized into three domains—an amino-terminal macro or X domain [41], a central alphavirus-specific region, and a carboxyl region with a hypervariable sequence containing several phosphorylation sites [42]. nsP3 residues located after the macro domain participate in the positioning of the P23 cleavage site [43]. nsP3 interferes with the formation of host cellular stress granules (SGs), which are involved in innate antiviral mechanisms, through the interaction of its carboxy-terminal domain with Ras-GTPase activating protein (GAP)-binding protein (G3BP) [44,45]. Finally, nsP4 is an RNA-dependent RNA polymerase involved in the synthesis of the different viral RNAs—namely, gRNA, sgRNA and minus-strand RNA complementary to the genome [46,47]. Preferential synthesis of the negative strand of viral RNA is accomplished by P123 + nsP4 complex, whereas nsP1 + P23 + nsP4 complex synthesize both positive and negative sense strands [48,49]. Fully mature nsPs are produced after the final cleavage event of P23, which switches the RNA template for synthesis of positive sense genomic and subgenomic RNAs.

Overall, SINV gRNA participates in three different functions: (1) As an mRNA to direct the synthesis of early viral nsPs; (2) as a template for the synthesis of the negative-strand RNA; and (3) by interacting with the capsid protein, it helps forming nucleocapsids during the assembly process to produce new virus particles. Furthermore, the negative-strand RNA serves as template to synthesize the two different viral mRNAs by the viral replicative machinery [49,50]. In SFV, genomic and negative-strand synthesis takes place within structures referred to as spherules, which are bulb-shaped invaginations of the membrane of virus–modified endosomes known as type I cytopathic vacuoles (CPV-1), which are induced after infection [34,51]. These spherules contain the replicative complexes and their size depends on the length of the replicated RNA [52,53]. Thus, the synthesis of viral RNAs in cytoplasmic RNA viruses takes place in close association with the cellular membranes [54,55]. The synthesis of cleavage intermediates of the alphavirus replicase can lead to membrane invaginations in the absence of viral replication. Thus, the formation of membranous spherules can occur in the absence of viral RNA synthesis [56]. Therefore, partially cleaved replicase proteins can participate in the assembly of replication complexes, membrane deformation, and in different stages of viral RNA synthesis. Analysis of the proteome of these replicative complexes has led to the identification of a number of cellular proteins that can up- or down-regulate their activity on RNA synthesis [57]. A number of host cellular factors can interact with nsPs, as has been shown for nsP2 and nsP3, and can modulate SINV RNA replication [58]. The recognition of an internal promoter in the negative strand RNA that is complementary to the gmRNA is necessary to initiate synthesis of sgRNA. This sgRNA is the most abundant SINV mRNA during the late phase of infection and directs the synthesis of five structural proteins initially as a polyprotein, C-E3-E2-6K-E1. Translation of this sgRNA is coincident with the dramatic inhibition of cellular mRNA translation.

**Figure 2:**
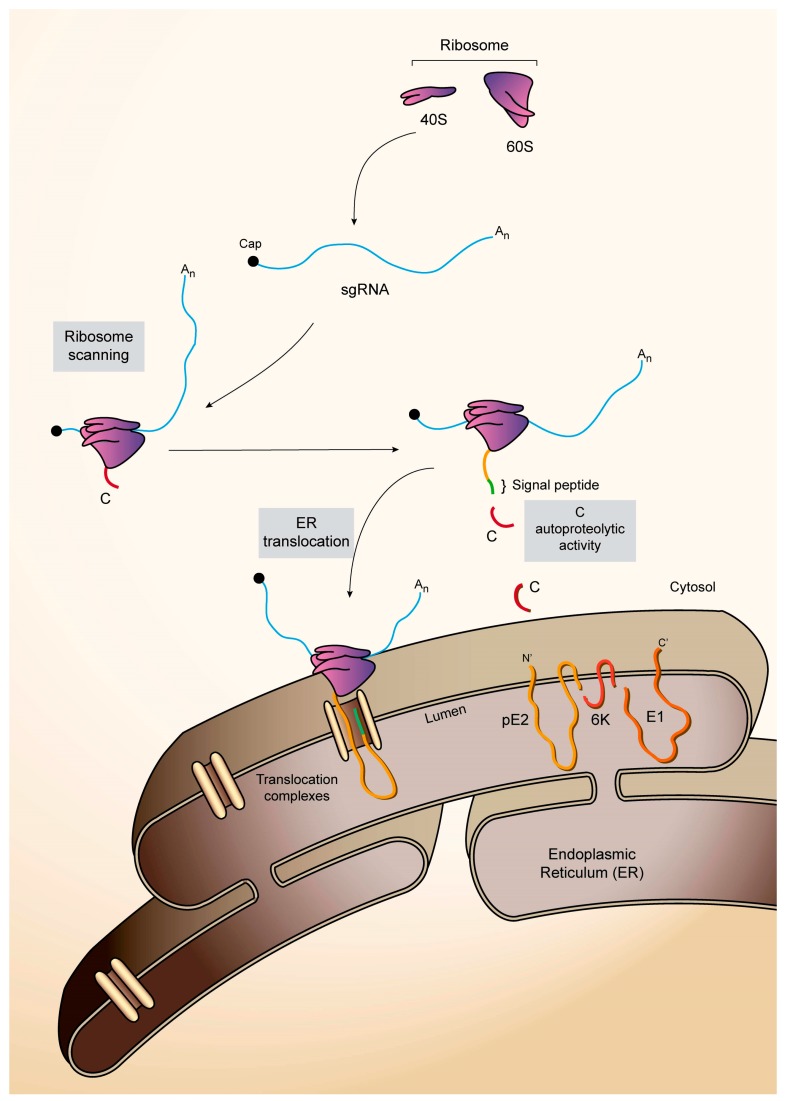
Schematic representation of SINV sgRNA translation to render structural proteins C (capsid)-E3-E2-6K-E1. The sgRNA coding sequence is flanked by two untranslated regions (UTRs): 5′-UTR, which contains a cap structure at its 5′ end, and 3′-UTR, which ends in a poly-(A) tail. The structural proteins are initially synthesized as a polyprotein. Ribosomes scan the capped sgRNA up to the first AUG and translation begins. First, C is synthesized and released from the polyprotein by autoproteolysis. The new N-terminus of the nascent polyprotein chain has a signal peptide for translocation to the endoplasmic reticulum (ER). Translation of the sgRNA continues, associated with the ER membranes, giving rise to the synthesis of the three glycoproteins E3, E2 and E1 and the viroporin 6K. The pE2 glycoprotein is synthesized across the ER membrane, where a carbohydrate attachment site may be responsible for the retention of the signal sequence in E2. The translocation of the glycoproteins across the ER membrane is regulated by various signal sequences. The glycoproteins and 6K are processed and cleaved by cellular proteases of the host vesicular system. Once the pE2–E1 heterodimer complex reaches the trans-Golgi, pE2 is cleaved by furin to form E3 and E2. The cleavage of pE2 is required to generate infectious particles.

The first protein to be synthesized during sgRNA translation is the capsid (C) protein, which is autocatalytically cleaved off the nascent chain upon translation of the polyprotein on the polysomes [11] (Figure 2). The C protein then binds to gRNA to form nucleocapsids in the cytoplasm. The amino-terminus of the E3 glycoprotein contains the signal peptide, which interacts with membranes of the endoplasmic reticulum (ER), and the polyprotein is translocated to the lumen. Here, it is cleaved by host cellular proteases, including furin and signalase, to render E3, E2, 6K and E1 proteins [59]. Some years ago, a heptanucleotide slip site (UUUUUUA) was discovered within the gene encoding 6K that, in about 10% of cases, results in the ribosome shifting to the −1 reading frame, rendering a novel transframe form (TF) of 6K and the E1 protein is not translated on these ocassions [60,61]. E2 and E1 interact with one and other to form dimers that migrate to the plasma membrane, leaving their carboxy-termini at the cytoplasmic face of the membrane. Nucleocapsids containing one copy of the genome interact with the cytoplasmic tails of viral glycoproteins to promote the budding of new virus particles [62]. At only 55 amino acids in size, the SINV 6K protein belongs to the viroporin family of proteins and is palmitoylated, helping it to target membranes [63,64,65,66,67]. The 6K protein is also involved in the transport of viral glycoproteins through the vesicular system to the plasma membrane [68,69]. As occurs with most viroporins, virus budding is promoted by 6K, but it is largely excluded from virions and is only detected in low amounts in mature virus particles [66,70]. By contrast, the transframe protein is apparently preferentially incorporated into released virions [71].

## 3. Inhibition of Host Translation by SINV Infection

Most cytolytic animal viruses induce a profound suppression of cellular protein synthesis in infected cells, particularly during the late phase of infection [72]. This inhibition would thus clearly interfere with the innate immune system and hence with the antiviral response [73]. This is the case for SINV, which blocks cellular translation in order to get the protein synthesizing machinery preferentially dedicated to translate the sgRNA, usually in a short time after infection (in BHK cells it occurs approximately 3 h after virus entry) but this process is dependent on cell line. A strong inhibition of host protein synthesis is found in vertebrate cells, but curiously, it is not observed when SINV infects mosquitos [74,75]. Therefore, it is likely that cellular and viral mRNAs are translated by different mechanisms. Although it is believed that gRNA is translated by a mechanism similar to that used for host mRNAs, we are still largely ignorant of the precise eukaryotic initiation factors (eIFs) necessary to initiate translation of SINV gRNA. In principle, both cellular and gRNA translation is down regulated at late phases of infection, when sgRNA directs the synthesis of structural proteins very efficiently [11]. The vast majority of cellular mRNAs contain a blocked cap structure at their 5′ end and are translated by the canonical cap-dependent scanning mechanism. This involves recognition of the cap by the heterotrimeric factor eIF4F, followed by the interaction of the preinitiation 43S complex with the mRNA [76]. The eIF4F complex is composed of the cap-binding factor eIF4E, the helicase and ATPase enzyme eIF4A, and the scaffolding protein eIF4G [77]. Unwinding of the secondary structure present in the mRNA leader sequence is accomplished by the preinitiation complex together with eIF4AI or eIF4AII, which are functionally interchangeable isoforms with 90% similarity [78]. After RNA unwinding, the 40S ribosomal subunit containing several eIFs linearly scans the leader sequence until an AUG codon is encountered in a good context [79]. Initiation of translation can also occur by other mechanisms independent of cap recognition, such as internal initiation. In this case, initiation takes place at an internal sequence located at the 5′ untranslated region (5′-UTR) of the mRNA, known as the internal ribosome entry site (IRES) [80,81]. Yet another mechanism of translation has been observed with SINV sgRNA, which contains a cap structure and is translated by a scanning mechanism of its rather short leader sequence without the participation of crucial eIFs such as eIF2 or eIF4A [82].

### 3.1. Mechanisms of Inhibition of Cellular Protein Synthesis by SINV Infection

Distinct mechanisms have been suggested to account for the abrogation of cellular protein synthesis by alphaviruses: (1) The phosphorylation of the α-subunit of eIF2; (2) Competition of viral mRNAs for the host translational machinery; and (3) Modifications of the cytoplasmic ionic environment. eIF2 plays a central role in mRNA translation and binds Met-tRNA_i_^Met^ and GTP to form a ternary complex that interacts with the AUG initiation codon and delivers the initiator Met-tRNA_i_^Met^ to the P site of the 40S ribosomal subunit. This event triggers GTP hydrolysis and eIF2-GDP is released to the cytoplasm to be recycled to eIF2-GTP by eIF2B. Phosphorylation of the α-subunit of eIF2 at serine 51 renders this factor inactive because it forms a stable complex with eIF2B and no recycling between GDP and GTP takes place [83,84]. Since the amount of eIF2B is about 10–20-fold less than eIF2, even a low percentage of eIF2α phosphorylation is sufficient to block the initiation of translation. eIF2 plays an important role in sensing metabolic status and cellular stress and, consequently, its activity is highly regulated by four known protein kinases that respond to distinct stress stimuli—protein kinase R (PKR) is activated by double-stranded RNA (dsRNA), PKR-like ER kinase (PERK) senses unfolded proteins in the ER, and GCN2 and heme-regulated inhibitor (HRI) are activated by nutrient starvation and heme deficiency, respectively [85].

SINV infection induces the phosphorylation of eIF2α in mammalian cells, which leads to an inhibition of host mRNA translation [86,87,88,89]. This is due to the activation of PKR by the synthesis of viral dsRNA in the cytoplasm [90]. Nevertheless, SINV infection of cells deficient in PKR, such as PKR^−/−^ murine embryonic fibroblasts (MEFs), also produce this blockade despite the fact that no increased eIF2α phosphorylation is observed [87,90]. Moreover, SFV infection can reduce the levels of phosphorylated eIF4E, the cap binding protein of the eIF4F complex; however, the significance of this finding is not clear [91]. It is possible that the lack of functionality of the eIF4F complex during infection leads to eIF4E inactivation.

The possibility that the translational efficiency and the quantity of sgRNA play a role in the inhibition of host protein synthesis has also been proposed [92]. Under this scenario, sgRNA would compete with cellular mRNAs for the translational machinery, which is logical since sgRNA is abundantly transcribed at late phases of infection and it is also efficiently engaged in translation [11]. However, SINV replicons encoding only for nsPs and lacking the coding sequences for sgRNA still induce a profound inhibition of cellular protein synthesis comparable to that observed in cells infected with wild-type virus [93,94]. Thus, in the absence of the synthesis of structural proteins directed by sgRNA, there remains a drastic suppression of host translation, pointing to the idea that competition is not necessary for this process. It is possible that the presence of abundant SINV mRNA sequences can interfere with host macromolecular synthesis, without participating directly in translation (see below). This type of competition may reflect the interaction with some cellular proteins by specific sequences of SINV mRNA, even in the absence of sgRNA translation. The imbalance of ionic concentrations in the cytoplasm of infected cells has been also implicated in the shut-off of host translation by several viruses, including SINV [95,96]. Indeed, at late stages of SINV or SFV infection, the ionic content of the cytoplasm is dramatically altered and plasma membrane permeability is increased [97,98]. This modification of the membrane is carried out by the 6K protein, which forms ion channels [66]. Yet, replicons that encode only for the capsid protein or for any structural protein fail to modify membrane permeability but still arrest cellular mRNA translation [88,94]. Overall, these observations suggest that SINV employs additional mechanisms to obstruct cellular protein synthesis.

### 3.2. Involvement of nsP2 in Host Translation Shut-Off

Because alphavirus replicons encoding solely nsPs obstruct cellular translation to a degree similar to that observed with wild-type virus, it was speculated that the synthesis of one of the nsPs was responsible for this inhibition [93]. Indeed, analysis of a number of alphavirus nsP variants pointed to nsP2 as being chiefly responsible for the inhibition of cellular macromolecular synthesis upon viral infection [37,90,99]. Accordingly, SINV with a single nsP2 point mutation at proline 726 presented defects in host translational shut-off [100]. Overall, these observations are consistent with the concept that nsPs are necessary to trigger the shut-off of host protein synthesis. However, mutations in the 5′-UTR sequence of SINV sgRNA leading to higher than wild-type levels of nsP2 were found to prevent the inhibition of host protein synthesis [92]. To reconcile these conflicting findings, we examined the inhibition of cellular mRNA translation mediated by individual nsPs and also by nsP1–4 [75]. We found that individual expression of nsP1, nsP2 and nsP3, or nsP1–4 had little effect on cellular protein synthesis. Of note, when nsP1–4 is expressed, not only are mature nsPs synthesized, but also their precursors, which is more akin to the situation observed in SINV-infected cells. As a control for these experiments, we expressed poliovirus (PV) 2A^pro^, which induces a profound arrest of cellular mRNA translation upon cleavage of eIF4G [101]. This result is in clear contrast to that found with SINV nsPs. Thus, the sole expression of nsPs is not sufficient to block cellular protein synthesis, and instead the strong replication of viral RNAs in the cytoplasm may be responsible for triggering this inhibition. In support of this concept, cellular shut-off does not occur in presence of inhibitors that reduce viral RNA replication [75]. Thus, treatment of SINV-infected baby hamster kidney (BHK) cells with two nucleoside analogs, 6-aza-uridine or ribavirin, prevents the inhibition of cellular protein synthesis even though sgRNA translation is still apparent. This prevention is not due to the inhibition of eIF2α phosphorylation, as it is also observed in PKR^−^/^−^ MEFs, which do not phosphorylate eIF2α after SINV infection.

### 3.3. Redistribution of Cellular Proteins between the Nucleus and Cytoplasm. A Proposal for the Mechanism of Cellular Translation Shut-Off

Several animal viruses provoke the relocalization of nuclear proteins to the cytoplasm as part of the cellular response to viral infection, leading to the formation of SGs [73,102]. Because a number of components that participate in protein synthesis are recruited to SGs, some viruses have evolved mechanisms to disrupt the formation of these inclusion bodies. Accordingly, SINV blocks SG formation by the interaction and complex formation of nsP3 with Ras-GTPase-activating protein SH3 domain-binding protein-1 (G3BP) [44,45]. Many of the nuclear proteins that are relocated to the cytoplasm after SINV or SFV infection are RNA-binding proteins (RBPs) and could directly interact with viral mRNAs, as has been found for T-cell restricted intracellular antigen-1 (TIA-1), heterogeneous nuclear ribonucleoprotein (hnRNP) A1, hnRNP K, hnRNP I, hnRNP M, polypyrimidine tract binding protein (PTB) or the ELAV RNA-binding protein HuR [75,86,103,104,105,106]. Indeed, HuR strongly interacts with the 3′-UTR of SINV and SFV mRNAs and participates in the regulation of their translation, transcription and replication [107]. Interestingly, sequences located at the 3′-UTR of SINV mRNAs are high-affinity binding sites for HuR, functioning with a “sponge”-like activity [108]. This sequestration of HuR on the cytoplasmic sgRNA has profound consequences for several cellular functions on host mRNAs, such as mRNA splicing, stability and decay.

As mentioned earlier, one plausible hypothesis to explain the shut-off of host translation is that viral RNA replication leads to high levels of viral sequences in the cytoplasm, that in turn induce the redistribution of nuclear proteins and the subsequent inhibition of protein synthesis. We found that the exit of nuclear proteins, including TIA-1 and PTB, is clearly detected in SINV-infected cells, but not upon the individual expression of nsPs or when viral RNA replication is reduced [75]. Moreover, the infection of BHK cells with the nsP2 P726G point mutation SINV variant, which exhibits defects in the shut-off of host protein synthesis, revealed that both viral RNA replication and the release of nuclear proteins to the cytoplasm are greatly inhibited. Thus, robust viral RNA replication must occur for the inhibition of cellular protein synthesis to proceed. Although this inhibition can take place via redundant mechanisms, such as redistribution of nuclear proteins, modification of eIFs, ionic imbalance or mRNA competition, it is probable that one of the most important factors to explain this event is the re-localization of nuclear proteins. In this regard, it is important to consider that a single viral protein that profoundly inhibits cellular translation, PV 2A^pro^, substantially modifies the shuttling of proteins between nucleus and cytoplasm. This viral protease not only cleaves eIF4G, but also several nuclear pore proteins (nuPs), disrupting the trafficking of proteins between nucleus and cytoplasm [109,110,111,112,113]. Based on this evidence, we propose that SINV replication leads to high levels of viral mRNAs in the cytoplasm that in turn modify the location of cellular proteins, triggering the blockade of host protein synthesis [75,92,108]. This can be accomplished either by the release of proteins that interact with cellular mRNAs or by sequestering components necessary for cellular translation on viral mRNAs due to their “sponge”-like activity [108]. Future characterization of the precise proteins that interact with viral and cellular mRNAs at late stages of infection in SINV infected cells should shed more light on this inhibition.

## 4. Structure of SINV sgRNA

**Figure 3:**
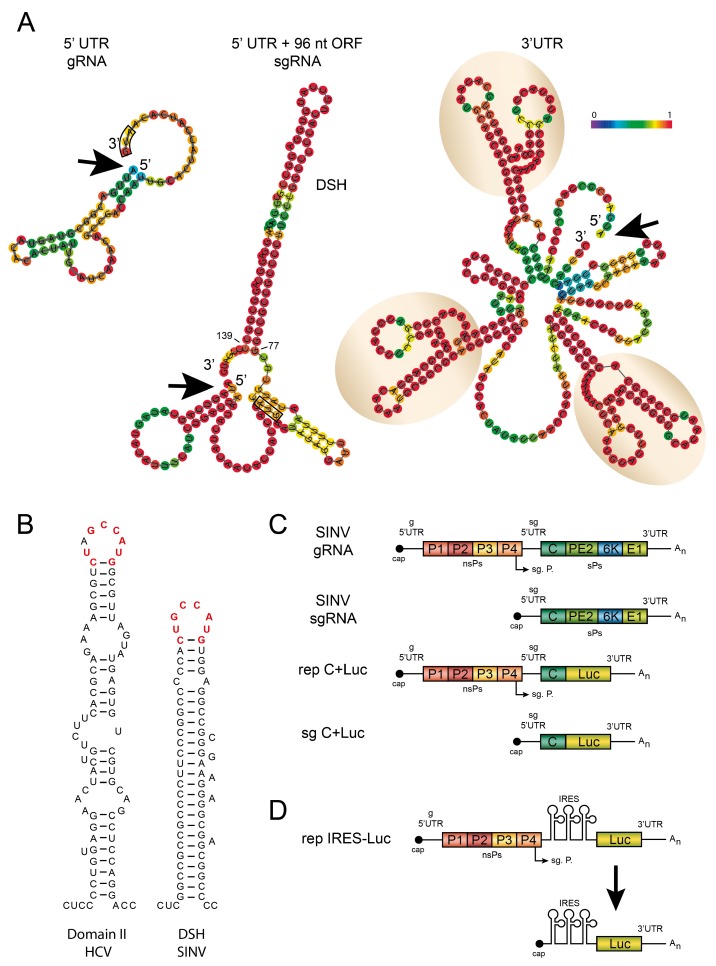
Some structural features of SINV mRNAs. (**A**) Secondary structure of the 5′-UTR and 3′-UTR regions of SINV gRNA and sgRNA. The 5′-UTR + 96 nt ORF of sgRNA include the downstream stable hairpin (DSH) from 77 to 139 nt. 5′-UTR gRNA has a free energy of the thermodynamic ensemble is −7.77 kcal/mol. 5′-UTR + 96 nt ORF sgRNA has a free energy of the thermodynamic ensemble is −60.31 kcal/mol. 3′-UTR has a free energy of the thermodynamic ensemble is −90.54 kcal/mol. These structures were obtained by The Vienna RNA Website. Nucleic Acids Res. 2008 (website tool: http://rna.tbi.univie.ac.at/cgi-bin/RNAWebSuite/RNAfold.cgi) and are colored by base-pairing probability. AUG start codons are shown surrounded by black boxes. Black arrows indicate the 5′ end of each RNA secondary structure. The three stem-loops at the 3′-UTR structure are highlighted within circles; (**B**) secondary structure of HCV Domain II and SINV DSH where similarity of the loops is marked in red; (**C**) schematic representation of SINV gRNA, sgRNA and the constructions encoding luciferase: (replicon) rep C+Luc and sgRNA C+Luc; (**D**) schematic representation of SINV construct containing the leader sequence of sgRNA replaced by an IRES element followed by the luciferase gene and the sgRNA synthesized from it. IRES: internal ribosome entry site.

A number of elements have been identified in sgRNA that make it particularly efficient for translation during infection (Figure 3). The SINV sgRNA is 4105 nucleotides (nt) in length without the poly(A) tail, and devotes the bulk of its sequence (3738 nt) to encode the structural polyprotein C-E3-E2-6K-E1. The coding sequence is flanked by two UTRs [114]. The 5′-UTR (49 nt) represents the leader sequence and contains a cap structure at its 5′ end. The 3′-UTR (323 nt) is organized in three different domains. In addition to the aforementioned 5′-and 3′-UTR, a hairpin (stem-loop) structure is present in the coding sequence at 77–139 nt from the 5′ end, which also participates in the translation of SINV sgRNA in infected cells.

### 4.1. The 5′-UTR of SINV sgRNA

Although the 5′-UTR leader sequence is rather short, it contains several motifs that are significantly implicated in different replicative functions, including transcription, translational shut-off, and viral pathogenesis (Figure 3A). The leader sequence contains a type 0 cap structure (N7mGppp) at its 5′-end, promoting RNA stability [114]. This leader sequence confers eIF4F complex-independence and is implicated in the inhibition of host translation [92,115]. Sequences in the negative-strand RNA complementary to the first 1–10 nt of the leader sequence are involved in promoter recognition and in the efficient transcription of sgRNA [114]. A SINV variant bearing a deletion of nucleotides 11–20 was found to be deficient for sgRNA transcription and failed to efficiently shut-off host cell translation, although it synthesized high levels of nsP2 [92]. By contrast, a revertant virus bearing a duplication of the promoter sequences was found to produce wild-type levels of sgRNA, and efficiently inhibited host translation. Accordingly, it has been postulated that this 11–20 nt region is necessary to interact with a cellular factor, which enhances viral translation and competes with cellular mRNAs [92].

The mechanism of the initiation of sgRNA translation and the selection of the AUG initiation codon have been studied in depth. Accordingly, sgRNA is translated via a scanning mechanism as the presence of a hairpin structure before the initiation codon hampers protein synthesis directed by this mRNA [82]. For this scanning process to occur, recognition of the cap structure by eIF4E is likely not necessary because cleavage of eIF4G by PV 2A^pro^ or human immunodeficiency virus (HIV) protease does not impede sgRNA translation in SINV-infected cells [88,115]. Moreover, this scanning on sgRNA takes place by a unique mechanism because it does not require some crucial initiation factors such as eIF2 and eIF4A [82].

### 4.2. The Hairpin Structure in the Coding Region of sgRNA 

Early studies identified sequences in the coding region of the SINV capsid protein that enhanced the translation of sgRNAs [116,117]. Prediction of a stem-loop structure in these sequences indicated the presence of a hairpin located 27 nt downstream of the AUG initiation codon, at position +1, the first adenosine of AUG. This downstream hairpin structure, previously termed the downstream loop (DLP) by us [87], is not a true enhancer of protein synthesis, but instead is involved in conferring eIF2-independent translation of sgRNA in infected mammalian cells [86,87]. A second important function of this DLP, better known as the downstream stable hairpin (DSH), is to signal the precise codon at which translation begins [88,117]. Thus, whereas disorganization of the DSH does not diminish translation in PKR-deficient MEFs, translation is obstructed when eIF2α is phosphorylated [86,87]. The DSH has been proposed to be responsible for adaptation to certain vertebrate hosts since no orthologue of the *PKR* gene has been found in insect cells [118]. A hypothesis has been put forward that the acquisition of the DSH structure has allowed the colonization of vertebrate hosts and the consequent geographic expansion of some alphaviruses worldwide [107].

An intriguing observation is that SINV sgRNA translation can occur even when the AUG codon is replaced by other codons [88]. For instance, the substitution of AUG by CUG, which encodes leucine, is particularly efficient as shown by the abundant amounts of structural proteins synthesized by this variant [119]. However, this phenomenon is not observed after disorganization of the DSH and a SINV variant sgRNA bearing CUG instead of AUG is practically unable to participate in translation if the DSH is disorganized [119]. Moreover, a loss of fidelity of sgRNA bearing genuine AUG is observed when the DSH is disorganized, leading to leaky scanning; in this scenario, the AUG initiation codon is not recognized in many initiation events and ribosomes pass through to select other downstream alternative AUGs [88,117]. The DSH therefore plays an important role in the selection of the start codon on sgRNA.

Much effort has been made to better understand the functioning of the DSH during the process of sgRNA translation. Based on the Kozak model, it was speculated that this hairpin stalled ribosomes leaving the AUG_i_ at the P site [117], thereby serving to mechanically stop the preinitiation complex in such a way that initiation at the AUG could be facilitated. This hypothesis, however, is unlikely because it is known that for a hairpin to facilitate initiation, it must be located 14 nt downstream of the AUG [79]. Placement of the DSH motif 9 nt closer to the AUG, which is an optimal position according to Kozak’s model at 15 nt from the AUG, strongly reduces translation. Moreover, we found that replacement of the DSH with a hairpin with a similar free energy does not confer translatability to sgRNA when eIF2 is phosphorylated [82]. In contrast to this “mechanical” model, we have proposed a “functional” action of DSH. Thus, its precise function would involve its active interaction with ribosomes, probably at the P site, in such a way to signal the correct codon and replace the activity of eIF2 [82]. It is also of interest to note that SFV sgRNA contains sequences that could interact with the 18S rRNA [120]. In conclusion, it is possible that the binding of DSH to ribosomes not only relieves the necessity for eIF2, but also signals the correct codon to initiate translation.

### 4.3. The 3′-UTR of SINV sgRNA

The SINV 3′-UTR is rather long (323 nt) and can be divided into three distinct regions (Figure 3A). A conserved 19 nt sequence can be found close to the poly(A) tail that, together with at least 11 nt of this tail, forms part of the promoter to synthesize minus-strand RNA [121,122]. An AU-rich sequence of about 60 nt is found before this conserved region, which interacts with the host protein HuR and is involved in mRNA stabilization during alphavirus infection [104,107,108]. Finally, there are three repeated stem-loop structures that are present not only in alphaviruses, but also in other arthropod-borne viruses (e.g., arboviruses) [122,123,124]. These elements, as well as the AU-rich domain, may contribute to the repression of deadenylation of viral mRNAs [125]. Deletion of most of the 3′-UTR whilst retaining the 19 nt conserved sequence decreases the efficiency of SINV replication in mosquito cells relative to chicken cells [126]. Moreover, mutagenesis of this region has different effects on viral replication in mice and in cultured murine cells [127], which are not only species-dependent, but are also dependent on the tissue analyzed. The alphavirus 3′-UTR thus has an important role in viral replication and adaptation to new hosts in mosquitos and mammalian cells. Indeed, adaptation to mosquitoes, rather than mammalian hosts, is a major evolutionary force on the CHIKV 3′-UTR. Deletions in the repeated stem-loop sequences result in the poor replication of the Asian lineages in mosquito vectors [128]. Overall, these findings indicate that this motif and the 3′-UTR play a significant part in the adaptation and evolution of CHIKV. We recently examined the role of the repeated stem-loop structure at the 3′-UTR of SINV mRNAs during the virus life cycle in mammalian and insect cells [129]. Notably, mutation of the three stem-loops had little effect on the translation of gRNA and sgRNA in mammalian cells; however, protein synthesis directed by these two mRNAs lacking this motif was profoundly suppressed in mosquito cells. Interestingly, the addition of the SINV repeated sequence elements to the short 3′-UTR of sleeping disease virus (SDV), an alphavirus that does not have an insect vector [130,131,132,133], potently increased its replication and translation in insect cells [129]. To our knowledge, this motif constitutes the first example of an element from an animal virus that confers translatability to mRNAs in a cell-specific manner and, accordingly, it could be described as a translation enhancer “cell-specific” element. These observations explain, at a molecular level, the acquisition of the repeated regions along alphavirus evolution. Indeed, it is thought that an alphavirus ancestor initially infected marine organisms and did not have an invertebrate vector [2]. Subsequently, the marine alphavirus ancestor adapted along evolution to infect invertebrate hosts by acquiring these repeated sequences at the 3′-UTR.

Another intriguing aspect of the three repeated stem-loops at the 3′-UTR is that any one of them could theoretically interact by base-pairing with a stem-loop close to the cap structure at the 5′-end (Figure 3A). It could be speculated that this base pairing is involved in sgRNA circularization to facilitate translation. However, mutations of the loop at the 5′-UTR or disorganization of the stem-loop have little effect on protein synthesis directed by sgRNA [129]. Thus, this interaction is perhaps important for virus replication at the organismal level. 

The 3′-UTR can also participate in the regulation of viral replication by its interaction with microRNAs (miRNAs), which regulate cellular protein synthesis through inhibition and/or degradation of mRNAs. The expression of miRNAs is cell specific and is regulated at the transcriptional level; therefore, the miRNAs present in a given tissue can also modulate viral replication [134,135,136]. One interesting example of this regulation of alphavirus replication is provided by the infection of hematopoietic cells by EEEV. The hematopoietic-specific miRNA, miR-142-3p, binds to specific sites at the 3′-UTR of the EEEV genome, blocking translation [137,138]. This inhibition in murine myeloid cells minimizes induction of type I interferon and other innate immune effectors, allowing EEEV to replicate almost undetected by host defense responses, which exacerbates disease in animal models. Removal of the miR-142-3p binding sites from viral gRNA rescues viral translation and replication in myeloid cells, resulting in enhanced systemic type I interferon production, prodromal signs of disease, and attenuation of the virus [137]. The potential role that endogenous miRNAs can play in the regulation of SINV replication is, however, not well understood. Indeed, human cell lines lacking a functional Dicer enzyme, and therefore unable to produce miRNAs or siRNAs, showed no enhancement in the replication of a variety of viruses including SINV [139], whereas deletion of the miRNA processing enzyme Drosha in mammalian cells led to higher viral replication [140]. Since the miRNA machinery naturally exerts an antiviral response in mammalian cells [141], the SINV-induced translocation of Drosha into the cytoplasm may represent a broad antiviral response.

## 5. Mechanism of SINV sgRNA Translation

Perhaps the most relevant aspect of sgRNA translation is that it can take place in the absence of several eIFs. The misleading concept that sgRNA translation can occur with “reduced levels” of eIFs suggested that this mRNA is translated using the eIFs necessary to translate cellular mRNAs, albeit at lower concentrations. In contrast to this notion, overwhelming evidence has shown that SINV sgRNA can be translated in the absence of active eIF4F, after efficient cleavage of eIF4G, or in presence of compounds that powerfully block the activity of eIF4A or eIF2. We recently showed in human cells that eIF2A and eIF2D do not participate in the initiation of protein synthesis directed by sgRNA [119]. Accordingly, SINV sgRNA has evolved novel sequences and structures for efficient translation during infection. It is fascinating that the evolution of these structures accommodates two different hosts—insects and vertebrates [129]; the final outcome being the generation of a viral mRNA that has eliminated the requirements for several eIFs.

A very interesting aspect of sgRNA translation is that it is tightly coupled to its transcription in infected cells [94]. Thus, transfection of sgRNA into cells at late stages of infection does not result in its translation, since only the sgRNA synthesized during viral transcription is recognized by the translational machinery. It is still not well understood why the transfected sgRNA, which contains all the elements for efficient translation, is excluded from the protein-synthesizing machinery. Ostensibly, only the newly-manufactured sgRNA at the viral replicative foci is engaged with ribosomes to direct protein synthesis.

### 5.1. Protein Synthesis Directed by sgRNA without an Intact eIF4F Complex

Early work with cultured cells doubly infected with PV and SFV indicated that the synthesis of structural proteins from alphaviruses was resistant, at least in part, to PV infection [54,142]. To examine this in more detail, we constructed SINV strains bearing the PV 2A^pro^ or the HIV *PR* gene under a second internal promotor. Infection of mammalian cells with these recombinant SINV strains led to the expression of PV 2A^pro^ or HIV PR and the consequent cleavage of eIF4G [115]. Under these conditions, no intact eIF4G was detected, but abundant synthesis of SINV structural proteins took place. Moreover, HIV PR not only cleaves eIF4G, but also poly (A)-binding protein (PABP) [143], suggesting that initiation on sgRNA does not require eIF4G or PABP. Overall, these results establish that an intact eIF4F complex is not necessary to initiate sgRNA translation, which begs the question of why this messenger contains a cap structure at its 5′ end but does not work as an IRES. A likely possibility is that other proteins or factors replace the recognition of the cap by eIF4E in the eIF4F complex. Indeed, this factor could be eIF3D, since it has been shown that it can participate in cap recognition on some specialized mRNAs without the requirement for eIF4E [144]. Moreover, eIF3 is necessary for initiation in vitro on sgRNA in reconstituted systems [145].

**Figure 4:**
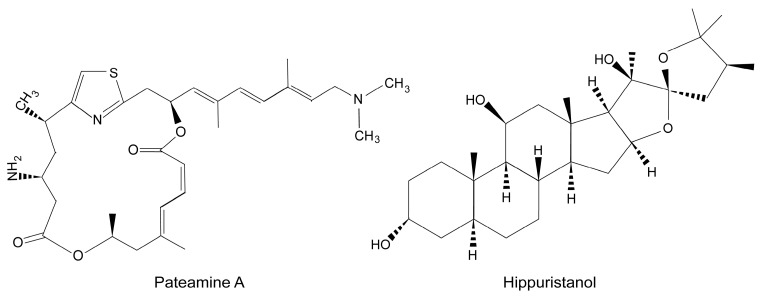
Chemical structure of pateamine A and hippuristanol.

Aside from the non-requirement of eIF4G, potent inhibition of eIF4A by selective inhibitors does not affect protein synthesis directed by sgRNA, reinforcing the concept that the eIF4F complex is not involved in the synthesis of SINV late proteins. In this regard, a number of new translation inhibitors have been discovered through high-throughput screening methods [146]. One such molecule is pateamine A (Pat A), a natural marine compound synthesized by the sponge *Mycale* sp. [147,148] (Figure 4). Pat A targets eIF4A and enhances its helicase and ATPase activities in vitro, leading to the disruption of its interaction with eIF4G and promoting the formation of a stable complex between eIF4A and eIF4B [149,150]. Thus, translation of capped mRNAs that require eIF4F is blocked. However, hepatitis C virus (HCV) mRNA is not inhibited by Pat A, although other mRNAs bearing picornavirus IRES elements are blocked by this compound [149,150]. Additionally, Pat A induces the formation of SGs by a pathway independent of eIF2α phosphorylation [151]. Protein synthesis directed by sgRNA is resistant to Pat A inhibition, whereas gRNA translation is blocked [152]. Interestingly, the resistance of sgRNA to Pat A is observed only in SINV-infected cells, and not when this mRNA is translated out of the virus replicative context. To our knowledge, this represents the first example of a capped mRNA that is resistant to Pat A.

A second potent and selective inhibitor of eIF4A is hippuristanol (hipp) [149,153] (Figure 4), a marine natural product isolated from the gorgonian coral *Isis hippuris* [146]. Hipp binds to the C-terminal domain of eIF4A, acting as an allosteric inhibitor of RNA interaction. This compound blocks translation of cellular mRNAs but not HCV IRES-driven translation. Notably, protein synthesis directed by sgRNA is not inhibited by hipp in SV-infected cells [74]; however, eIF4A is required to translate this mRNA in transfected cells or in cell-free systems. Perhaps, the modifications of the cytoplasm in the infected cells may create an environment that modifies the requirements for eIFs in the translation of sgRNA.

### 5.2. Translation without eIF2

**Figure 5:**
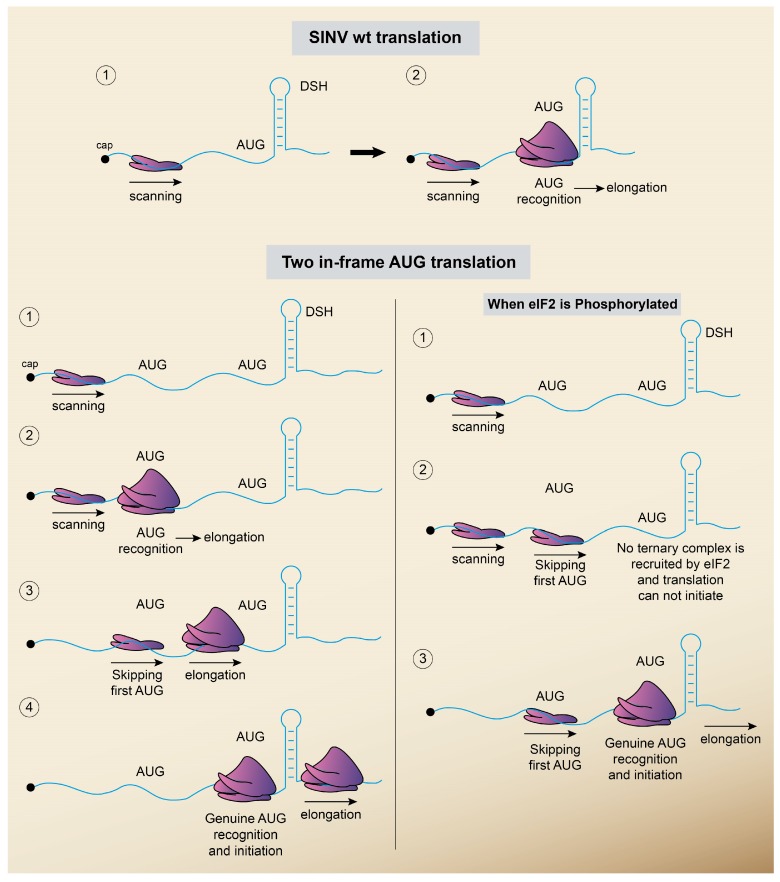
Schematic representation of the model for the initiation of translation on SINV sgRNA. (Upper panel) In the model of the scanning mechanism followed by WT SINV sgRNA to initiate translation, the 40S ribosomal subunit attaches initially at the 5′ cap structure. Then, the 5′ UTR is scanned base-by-base in a 5′–3′ direction until the initiation codon (AUG) is recognized. (Lower panel) Model for translation initiation on SINV sgRNA bearing two alternative start codons (AUG). Under no stress conditions (left part), the preinitiation complex containing the 40S ribosomal subunit interacts with the cap structure and scans the leader sequence of sgRNA until the first AUG is encountered. Then, the 80S initiation complex can be formed and elongation ensues. Another preinitiation complexes can start scanning from the cap structure and, in some cases, skip the first AUG start codon and reach the second AUG (genuine AUG), initiating the synthesis of authentic C protein from this start codon. When eIF2 is phosphorylated (right part), the lack of functional eIF2 prevents the initiation in the first AUG, nevertheless the genuine AUG, which is in proximity with DSH, manages to initiate the translation independently of the eIF2.

As mentioned earlier, SINV infection induces the phosphorylation of eIF2α leading to its inactivation via activation of PKR by dsRNA. Several inhibitors such as sodium arsenite, dithiotreitol or thapsigargin can further increase this phosphorylation in SINV-infected cells, from about 80% in untreated cells to virtually 100% eIF2α phosphorylation in treated cells [88]. This finding demonstrated that sgRNA translation did not occur at reduced levels of active eIF2, but rather took place when practically all eIF2 was inactivated. As discussed previously, the hairpin located between 27 and 89 nt downstream from the AUG initiation codon is crucial to translate this mRNA when eIF2α is inactivated [74,86,87,89]. It could be speculated that although the majority of eIF2α is phosphorylated in SINV-infected cells, a small portion of active eIF2 remains in close proximity to the translation machinery at sgRNAs. We evaluated this possibility by generating specific variant SINV sgRNAs containing two in-frame AUG start codons [82] (Figure 5). Curiously, initiation on this artificial sgRNA took place at both AUGs, but each of them was preferentially selected depending on the activity of eIF2. Thus, after eIF2α phosphorylation, translation on one AUG was reduced, while the initiation codon closest to DSH was resistant to this inhibition. This result shows that on a single mRNA, one AUG requires active eIF2 whereas the second one, which is at a short distance to the first AUG, initiates translation in an eIF2-independent manner.

We have previously proposed that the function of eIF2 in SINV-infected cells can be replaced by other cellular factors, such as eIF2A [87]. eIF2A was described several years ago, but its precise activity in mammalian cells remains unclear and deletion of the yeast orthologue has no effect on cell viability [154]. Early results demonstrated that eIF2A can interact with Met-tRNA_i_^Met^ to bind it to the ribosome [155]; however, this binding was much less efficient than that observed using genuine eIF2 on artificial templates. Moreover, eIF2A was unable to promote the binding of Met-tRNA_i_^Met^ to globin mRNA [156]. Recent findings suggest that eIF2A is involved in the translation of some specialized cellular mRNAs that initiate translation with non-AUG codons [157,158]. Of interest, eIF2A has been implicated in cancer progression because it is involved in the initiation of translation of unconventional upstream ORFs [159]. Surprisingly, the development of mice with deletion for the *eIF2A* gene is completely normal, indicating that *eIF2A* is not required for the translation of both normal and specialized cellular mRNAs [160]. Another possibility is that *eIF2D* initiates sgRNA translation in place of eIF2 [145]. eIF2D was initially purified from rabbit reticulocyte lysates as an activity that could displace deacylated tRNA and mRNA from recycled 40S ribosomal subunits. In addition, eIF2D could interfere with the formation of the 48S initiation complex promoted by eIF2 [145]. A complex between Met-tRNA_i_^Met^ and eIF2D is formed in a GTP-independent fashion, and can interact with the 40S ribosomal subunit to deliver the initiator to the ribosomal P site [161]. However, as with eIF2A, the precise function of eIF2D in mammalian cells remains enigmatic.

To test the potential role of eIF2A and eIF2D in translation, we used human wild-type haploid HAP1 cell lines or equivalent cells knocked-out for eIF2A, eIF2D or both by CRISPR/Cas9 genome engineering. Cellular morphology, global protein synthesis and SINV infection was comparable between all four cell lines [119]. Moreover, synthesis of viral proteins at late stages of infection also was similar despite the fact that eIF2α became phosphorylated [119]. These findings show that eIF2A and eIF2D are not required for the translation of sgRNA when eIF2α is phosphorylated. Moreover, silencing of eIF2A or eIF2D by transfection of the corresponding siRNAs in HAP1 WT, HAP1-eIF2A^−^ and HAP1-eIF2D^−^ cells had little effect on the synthesis of viral proteins late in infection. Elegant studies employing CRISPR/Cas9 technology in HAP cells has provided an extensive analysis of the human proteins that are involved in the replication of HCV, and are dispensable for cell viability [162]. Curiously, some of these proteins are not required for SINV replication. These results provide an interesting approach to develop antiviral compounds against human viruses.

Our observations support the novel proposal that eIF2 is not replaced by a cellular protein during the translation of SINV sgRNA. Instead, this viral mRNA has evolved a specialized structure that confers independence for eIF2. In this regard, it is possible that the DSH functions in a way similar to that of domain II of HCV IRES, because there are also great similarities between these elements, including the sequence present at the loop (Figure 3B). Thus, domain II from HCV IRES can directly interact with preinitiation complexes and 80S ribosomes and displace the bound eIF2, substituting the requirement for this factor [163,164]. It can be hypothesized that some viral mRNAs can acquire elements to maximize the translation process under infection conditions. The consequences for the virus life cycle are that significant amounts of structural proteins can be produced upon the translation of sgRNA even under stress conditions that appear after viral infection.

### 5.3. Mechanism of sgRNA Translation. Comparison with Cellular mRNAs

Work carried out in the past few years has provided a more detailed picture on the mechanism by which sgRNA initiates translation. To compare this initiation mechanism with the canonical cap-dependent scanning mechanism that takes place on cellular mRNAs, we will briefly summarize the molecular events that initiate translation on cellular mRNAs. Cap recognition involves the interaction of eIF4E with the methylated structure m7GpppN located at the 5′ end of eukaryotic cellular mRNAs [77]. Binding of eIF3 to the eIF4G middle domain promotes the interaction of the preinitiation complex 43S at the 5′ end of mRNAs [165]. Thus, there is an interaction of the ribosomal subunit 40S, containing several eIFs such as eIF1, eIF1A, eIF3, and eIF2 in the form of ternary complex [166,167]. It has been proposed that the 40S ribosomal subunit bound to these eIFs is in an “open” conformation, that is, competent for scanning, which involves linear base-by-base inspection of the 5′-UTR [79,166,168,169]. This scanning takes place until an AUG initiation codon is found in a suitable sequence context [79,170]. The secondary structure of the 5′-UTR is melted during the scanning process, in part by the helicase activity of eIF4A, although stable hairpins cannot be melted by small ribosomal subunits and eIF4A [78]. After positioning of the preinitiation complex at the AUG initiation codon, base-pairing takes place with the anticodon present in the initiator tRNA Met-tRNA_i_^Met^. Subsequently, the eIF5 carboxy moiety promotes the dissociation of eIF1, together with inorganic phosphate derived from the GTP hydrolysis of the ternary complex [171,172]. In addition, the eIF1A carboxy terminus moves closer to the eIF5 amino terminus [173]. This movement is coupled to eIF1 exit, which leaves the P site free and allows tighter binding of the initiator tRNA at this site. Concomitant with this rearrangement, eIF5B-GTP can now interact with the 40S subunit. In this manner, eIF5 together with eIF2-GDP are released from the small ribosomal subunit, which is now in the “closed” conformation and is committed to continue mRNA translation [172,173]. This interaction of eIF5B-GTP stimulates the joining of the 60S subunit to form an 80S initiation complex. The initiation phase ends with the Met-tRNA_i_^Met^ accommodated in the P site of the 80S, leaving free the A site. The interaction of the ternary complex aminoacyl-tRNA-eEF1-GTP to this site starts the elongation phase.

In common with cellular mRNAs, the initiation of SINV sgRNA translation also takes place following the scanning mechanism [74,82]. The first event in this initiation could be the interaction of eIF3, by means of its subunit eIF3D, to the cap structure at the 5′-end, without the participation of eIF4E and the entire eIF4F complex [115,144,152]. After the interaction of eIF3 with the cap structure, the 40S ribosomal subunit can bind to the mRNA. Although exactly which eIFs bind to this 40S ribosomal subunit remain unclear, the ternary complex containing active eIF2 is definitely not required for this interaction, nor for the subsequent scanning of the leader sequence [129]. Once the 40S in the “open” conformation (with some still undefined eIFs) reaches the AUG initiation codon, it can stop to interact with the stable hairpin loop that could bind to the ribosomal P site, inducing the “close” conformation. This event would promote the joining of the 60S subunit to build-up the 80S ribosome competent to translate this viral messenger. How the Met-tRNA is delivered to the ribosome in order to establish the codon-anticodon based pairing remains unknown. The finding that other codons can replace AUG to initiate translation of sgRNA, albeit with lower efficiency, makes it possible that aminoacyl-tRNAs different from Met-tRNA can participate in this process [88,119]. Since active eIF2 is not required for this initiation event, perhaps other cellular factors, including elongation factor eEF1, are responsible for this event following a mechanism akin to that reported for cricket paralysis virus (CrPV) [174,175]. The similarities in the structure between the DSH and domain II of HCV IRES points to the possibility that their functioning is also similar (Figure 3B) [163,164], since translation of HCV is independent of active eIF2. Once the 80S ribosome has been built-up, it can initiate the elongation phase to synthesize the polyprotein that contains the SINV structural proteins. A puzzling aspect of sgRNA translation is that the 80S has to pass through the DSH, melting its structure. Clearly the DSH cannot be functional during the translation of this hairpin because it would remain disorganized. After the ribosome passes, the hairpin must reorganize to become functional on the 40S ribosomal subunit that is paused at the start position. Therefore, the DSH is melted and reorganized each time that the ribosome translates this sequence of the capsid protein. The obvious question that arises is why the DSH is located at the coding region and not at the leader sequence. A possible reason could be that since the mRNA is capped, the 40S ribosomal subunit could not melt this structure and necessarily should be placed after the initiation codon at an optimal distance to exert its function. It must be considered that the preinitiation complexes containing the small ribosome subunit are unable to melt the DSH hairpin, and only 80S ribosomes have the potential to pass through this stem-loop.

## 6. Translation of SINV sgRNA Bearing IRES Elements

The singular translation system represented by SINV-infected cells provides a good model to analyze the requirements of some specialized mRNAs to direct protein synthesis. This is because high amounts of sgRNA are present at late stages of infection and this is the only mRNA efficiently translated after the abrogation of cellular protein synthesis. Against this background, the translatability of sgRNAs bearing different IRES elements has been studied in mammalian cells using SINV replicons, and a number of surprising results, some of them remaining unresolved, have been reported during IRES-driven translation in SINV-replicating cells [89,176].

### 6.1. The Variety of Internal Ribosome Entry Site Elements 

There is a great variety of IRES elements with regards to their structure and functioning, and several have been analyzed in cellular and viral mRNAs [177,178,179]. In the case of animal viruses, four major groups are known to contain mRNAs bearing IRES elements: picornaviruses, flaviviruses, pestiviruses and retroviruses [178,180,181,182,183]. Picornavirus IRES elements can be classified into at least two groups—IRES type I is typical of entero/rhinoviruses, with PV considered as the prototype, whereas type II IRESs are present in cardio/aphtoviruses, with encephalomyocarditis virus (EMCV) as the prototype [181,184,185]. IRES elements contain a rich secondary structure with several stem-loops, which are crucial for their activity. Most IRES elements bear a tRNA-like motif that is involved in binding to ribosomes [186,187,188]. The requirement for eIFs varies according to the IRES under study. Thus, picornavirus IRESs do not require eIF4E and can be translated when eIF4G is cleaved by some picornavirus proteases, such as PV 2A^pro^ or foot-and-mouth disease virus (FMDV) leader (L)^pro^ [81,189,190]. Notably, HCV mRNA can be translated without the eIF4F complex and even in the absence of eIF2 [187]. Perhaps most strikingly, the intergenic region (IGR) of CrPV mRNA directs protein synthesis in the absence of all known initiation factors [182]. In addition to eIFs, a number of cellular proteins known as IRES trans-activating factors (ITAFs) have the ability to interact directly with IRESs and modulate their activity [183,191,192].

### 6.2. IRES-Driven Translation in Alphavirus Replicons

In the early days of research on alphavirus translation, it was discovered that gRNA only directed the translation of the first ORF, whereas those proteins encoded by the second cistron were synthesized from a second mRNA (sgRNA) [193]. The implication was that only the AUG initiation codon nearest to the cap structure was functional, and not the internal AUG present in sgRNA. Therefore, the leader sequence of sgRNA was thought to have no IRES activity. Indeed, transfection of uncapped gRNA in mammalian cells failed to direct the translation of a reporter gene located in the second cistron [176]. It is appreciated that mRNAs containing IRES elements are very efficiently translated, both in cultured cells and in in vitro systems, and for this reason a number of viral vectors have been developed bearing IRES elements to provide robust gene expression. In this regard, alphavirus vectors are potentially useful tools to express heterologous genes and for the design of vaccines [194,195,196]. However, the use of IRES elements in alphavirus vectors results in poor gene expression [176,197]. Indeed, IRES elements belonging to picornaviruses HCV and CrPV perform poorly in SINV replicating cells [89,176]. SINV constructs containing the leader sequence of sgRNA replaced by an IRES element followed by the luciferase gene are able to synthesize luciferase protein when uncapped gRNA is transfected into mammalian cells, demonstrating that internal initiation on gRNA occurs early after transfection (Figure 3D). Notably, the translation is inhibited in the late phase of SINV replication. Co-expression of different PV non-structural genes has revealed that PV 2A^pro^ can increase translation of sgRNAs containing the PV or EMCV IRESs, but not of those from HCV or CrPV. The L^pro^ protease from FMDV also rescues translation, whereas a PV 2A^pro^ variant deficient in eIF4G cleavage does not increase picornavirus IRES-driven translation in SINV replicons. Overall, these findings suggest that the replicative foci of SINV-infected cells, where sgRNA translation takes place, are deficient in components necessary to translate IRES-containing mRNAs. In the case of picornavirus IRES elements, cleavage of eIF4GI by PV 2A^pro^ or FMDV L^pro^ rescues this inhibition. The fact that translation of picornavirus IRESs requires functional eIF2 at early stages of infection, but not later [198], suggested that the lack of picornavirus IRES-driven translation in SINV-replicating cells was due to the phosphorylation of eIF2. Indeed, PV IRES-driven translation can take place from SINV replicons if eIF2α remains unphosphorylated in PKR^−^/^−^ MEFS [89]. It was therefore concluded that these viral proteases conferred eIF2-independent translation to picornavirus IRESs [89,189,190,199]. Thus far, no explanation has been proposed for the failure of HCV or IGR CrPV IRES elements to direct protein synthesis in SINV replicating cells. In the case of HCV IRES, it is independent of both the eIF4F complex and eIF2, a situation similar to that described for sgRNA translation [200]. Even more intriguing is the fact that IGR CrPV IRES, which does not require any eIF [175,201], is inactive in the context of SINV replicons [176]. Perhaps these IRES elements require an ITAF that is not present in the replicating foci of SINV-infected cells. Alternatively, it is possible that the redistribution of nuclear proteins to the cytoplasm is inhibitory for the translation of these IRES elements. This latter possibility is more likely given that all the viral IRES elements are functional on uncapped gRNA early after transfection.

## 7. Concluding Remarks and Future Perspectives

The study of the regulation of protein synthesis in SINV-infected mammalian and insect cells has broadened our understanding of the basic translation mechanisms of viral mRNAs. In this regard, several elements have been identified in SINV mRNAs that maximize their translatability in different host cells. These viral messengers have thus exhibited functional plasticity during evolution to adapt to different species and environments. Most probably, alphaviruses first appeared in marine vertebrates [2] and expanded their host range by acquiring the ability to infect insects, which became effective vectors for viral transmission to terrestrial vertebrates. In the adaptation to insects, alphaviruses recruited a motif at their 3′-UTR, which is necessary for the powerful translation of their mRNAs in this host. However, the precise functioning of this motif as regards to its interaction with cellular factors remains enigmatic. Although we now comprehend the functioning of DSH and the leader sequence of these mRNAs, additional efforts are needed to better understand their activity during the initiation of translation. In particular, further work is necessary to address the mechanism of translation of SINV gRNA, to discern if it follows the canonical pathway exhibited by cellular mRNAs, and also the explicit eIFs involved in the initiation of protein synthesis. Finally, the abrogation of host protein synthesis after infection of vertebrate cells by SINV would appear to be due to the redistribution of nuclear proteins to the cytoplasm. However, we do not know which RNA-binding proteins interact with viral and cellular mRNAs at late stages of infection. Comprehensive proteomic analysis will be essential to identify which cellular (or viral) proteins interact with mRNAs and may shed more light on the control of host cell translational machinery by SINV. If translational shut-off is indeed due to the accumulation of nuclear proteins in the cytoplasm of infected cells, several questions will need to be addressed: (1) Which nuclear proteins are able to interact with cellular mRNAs and, from those, which ones are responsible for the inhibition of protein synthesis? (2) Why is this blockade selective for host cell translation? (3) Is the presence of viral RNA sequences responsible for the relocalization of nuclear proteins to the cytoplasm following the “sponge-like” mechanism and consequently for the inhibition of host translation? Future work using the SINV infection model will answer some of these questions.
